# Differentiation *of Basidiobolus* spp. Isolates: RFLP of a Diagnostic PCR Amplicon Matches Sequence-Based Classification and Growth Temperature Preferences

**DOI:** 10.3390/jof7020110

**Published:** 2021-02-03

**Authors:** Maike Claussen, Stefan Schmidt

**Affiliations:** School of Life Sciences, Discipline of Microbiology, University of KwaZulu-Natal, Pietermaritzburg 3201, South Africa; schmidts@ukzn.ac.za

**Keywords:** *Basidiobolus*, differentiation, growth temperature, diagnostic PCR, restriction analysis, RFLP, 28S rRNA, zygospore

## Abstract

The genus *Basidiobolus*, known since 1886, is primarily associated with reptiles and amphibians. Although globally distributed, rare infections caused by members of this genus mainly occur in tropical and subtropical regions. Morphological and physiological characteristics were used in the past for the description of species. However, some of these characteristics vary depending on culture conditions. Therefore, most species names are regarded as synonyms of *B. ranarum* as the only pathogenic species. Yet, not all environmental isolates are necessarily pathogenic. This study aimed to analyze if environmental *Basidiobolus* isolates can be distinguished reliably based on morpho-physiological and molecular characteristics. Eleven isolates originally obtained from feces of south African reptiles and one type strain, *Basidiobolus microsporus* DSM 3120, were examined morpho-physiologically. Sequence analysis of the 18S and partial 28S rRNA gene and restriction analysis of a diagnostic amplicon (restriction fragment length polymorphism, RFLP) were performed for all 12 strains. Based on the results obtained, morphological features and the 18S rRNA sequence proved insufficient for the reliable differentiation of isolates. However, isolates were distinguishable by growth temperature profiles, which matched isolate clusters established by partial 28S rRNA gene sequence and restriction analysis of a *Basidiobolus* specific diagnostic PCR amplicon. Our results indicate that RFLP analysis can be used as a fast screening method to identify *Basidiobolus* isolates with similar physiological characteristics.

## 1. Introduction

Members of the filamentous fungal genus *Basidiobolus* are found worldwide associated with amphibians, reptiles, as well as other animals, soil, and plant detritus [1,2,3,4,5,6,7,8,9,10,11,12]. However, infections in animals, including humans, are rare and mainly reported for warmer subtropical to tropical regions [13,14,15,16,17,18,19,20,21]. The first species of this genus was described and isolated in 1886 by Eidam from frog excrements and named *Basidiobolus ranarum* [1]. Later, additional species such as *B. haptosporus*, *B. meristosporus*, *B. microsporus*, *B. magnus*, and *B. heterosporus* were isolated from various animals and environmental sources [2,3,4,22,23,24]. In the past, morphological features such as the formation of undulated versus smooth zygospores were used for differentiation along with physiological characteristics such as temperature requirements for growth and odor production [3,22,23,24,25]. However, the reliable differentiation of *Basidiobolus* species by morphological and physiological characteristics proved to be difficult as variations between strains of the same species and changes of such characteristics during cultivation were reported [13,26,27,28,29,30]. So far, only *B. microsporus* possesses a unique morphological characteristic: the formation of exogenous microspores [3,25,29,31]. This separation of *B. microsporus* from all other *Basidiobolus* species is supported by studies comparing exoantigens [32], isozyme variation [33], and “rDNA” analysis [34]. 

The ongoing controversial discussion about the taxonomy of *Basidiobolus* species resulted in the suggestion that only one species name, *Basidiobolus ranarum*, should be used for the pathogenic species, and other previously used species names such as *B. meristosporus* or *B. haptosporus* should be considered as synonyms [31]. Currently, it is assumed that only *B. ranarum* can cause infections [19,21,35], although other *Basidiobolus* species are still frequently reported in the literature. The use of synonyms can lead to confusion when new isolates are described. Furthermore, assigning new *Basidiobolus* isolates without proper characterization to only one species, namely *B. ranarum*, does not help either as not all environmental strains might at the same time be potentially pathogenic. Unfortunately, and complicating matters further, no type strain cultures of the original species *Basidiobolus ranarum* Eidam, which could be used as reference, appear to exist in any of the well-known culture collections (e.g., ATCC, CBS). However, type strains for species such as *B. heterosporus*, *B. meristosporus*, and even *B. magnus* are available in culture collections. The availability of sequencing technologies provided new opportunities to compare and differentiate microbial isolates. Indeed, the analysis of sequence data from various *Basidiobolus* spp. isolates, including type strains, revealed some genomic differences between species, with six *Basidiobolus* species assumed [36,37,38,39]. Still, for a proper characterization of isolates, genomic sequence analysis alone is not sufficient given that growth characteristics and other physiological parameters cannot be predicted that way reliably. Hence, physiological tests are still an essential part of the proper characterization of fungal isolates. Therefore, fungal identification should include a combination of molecular, physiological, and morphological analysis [40].

PCR-based methods for a reliable identification of the genus *Basidiobolus* are available [41,42,43,44] and are a useful tool for verifying and confirming microscopical and culture-based identification. Yet, as these PCR-based methods do not distinguish between possible species or subspecies, it is not certain if only one or more than one species or subspecies are responsible for an infection and how these different strains are distributed in the environment.

Therefore, we investigated if *Basidiobolus* spp. isolates originally obtained from reptile feces in South Africa could be divided into distinct groups using selected morphological and physiological characteristics and if this grouping could be confirmed using molecular methods. Additionally, we examined if restriction fragment length polymorphism (RFLP) analysis of a group-specific diagnostic PCR amplification product (Ba1/Ba2 [41]) can be used to distinguish these environmental isolates accordingly. This would allow for the screening of large numbers of isolates, confirming membership of the genus *Basidiobolus* and at the same time assignment to groups of related strains without using extensive and time-consuming cultural methods. 

## 2. Materials and Methods 

### 2.1. Basidiobolus Isolates

A total of 12 *Basidiobolus* strains was used for morphological, physiological, and sequence-based characterization (Table 1). Nine *Basidiobolus* spp. isolates were obtained in a previous study [12] analyzing reptile feces from a suburban area in Pietermaritzburg (KwaZulu Natal, South Africa). Additionally, two isolates (Cla6, C3-1) were obtained from gecko feces collected in Clarens (Free State, South Africa) and identified as described previously [12]. The type strain *Basidiobolus microsporus* DSM 3120 was used for comparison. All isolates were kept as living cultures on non-selective media such as Sabouraud Dextrose, Nutrient, and Wort Agar at ambient temperature with regular sub-culturing for maintenance. All microscopic analyses were done using a Zeiss Axio Scope with an Axiocam ICc3. Images were taken using Zen 2.3 blue edition and assembled with Affinity Photo (version 1.8.5.703).

### 2.2. Effect of Temperature on Growth 

The radial growth of all 12 strains was determined on Sabouraud Dextrose Agar (SDA; Neogen, Lansing, MI, USA) at six different temperatures (6, 20, 28, 37, 40, and 45 °C). For inoculation, all isolates were grown on Nutrient Agar (NA; Neogen, Lansing, MI, USA) at 28 °C for 4 to 7 days until sufficient growth (clear mycelia formation) was visible. Plugs of 6 mm diameter were aseptically taken from the mycelial growth perimeter of these cultures and placed onto SDA. Radial growth at the different temperatures was measured to the nearest millimeter (maximal diameter) every 24 h for 96 h and again after 7 days (at least 6 measurements from 3 independent experiments). If no growth was detectable after 7 days, the incubation was extended, and additional radial growth measurements were taken after 10 and 14 days. The presence or absence of growth was confirmed microscopically (hyphae formation).

### 2.3. PCR and Sequence Analysis

Total DNA was isolated using the *Quick*-DNA Fungal/Bacterial Microprep kit (Zymo Research, CA, USA) according to the manufacturer’s instructions. PCR reactions for DNA amplification included an initial denaturation at 94 °C for 5 min, followed by 35 cycles consisting of primer specific denaturation, annealing, and extension steps, and a final extension at 72 °C for 7 min. All primers and cycling conditions used are given in Table 2, and the primer sequences are specified in Table S1. 

Typical PCR reactions were performed in a final volume of 26 μL containing 2× PCR reaction mix (OneTaq, NEB, MA, USA), 11 pmole of each primer, and 0.5 μL of DNA. Nuclease-free water was used as the negative control. The presence of amplification products was confirmed by gel electrophoreses (1.5% agarose gel, 0.5× TAE-buffer, ethidium bromide staining). Bidirectional sequence analysis of amplification products obtained with the primer pairs 3–6 (Table 2) was performed by the CAF DNA Sequencing Unit (University of Stellenbosch, South Africa).

Sequence data obtained with primer pairs 3 to 5 (composite 18S rRNA gene region) and 6 (D1/D2 domain of 28S rRNA gene region) were assembled using Geneious prime (version 2020.2). Consensus sequences obtained were deposited with GenBank under the accession numbers shown in Table 1. Comparison with deposited sequences in GenBank was done using the nucleotide basic local alignment search tool BLAST (Megablast, accessed on 26 August 2020). Phylogenetic trees were established using Geneious Prime (version 2020.2) with alignments done using Clustal Omega and using sequences of type strains deposited in GenBank for comparison. As no type strain sequence is available for *B. ranarum*, a sequence from a certified reference strain (ATCC 14449) was used instead.

### 2.4. Restriction Analysis

The sequences for the PCR amplicon products using the diagnostic primer pair Ba1/Ba2 (28S rRNA) were extracted from the results obtained from 28S rRNA gene sequence analysis for each isolate and examined for suitable enzyme restriction sites for the differentiation of isolates. To confirm the predicted restriction pattern, the group-specific Ba1/Ba2 PCR amplification product was used. Fast digestion of the PCR products was performed at 37 °C for 2 h with HaeIII and HinP1I (Fermentas) and for 4 h with AccI (NEB), respectively, followed by a final incubation at 80 °C for 10 min to stop the reaction. Restriction took place in a total reaction volume of 15 μL using an enzyme/PCR product ratio following the manufacturer’s recommendations. Restriction fragments were separated in a 2.5% agarose gel for 1 h at 200 V using 1× TB buffer [49] followed by ethidium bromide staining. 

## 3. Results

### 3.1. Morphological and Physiological Characterization

**Figure 1:**
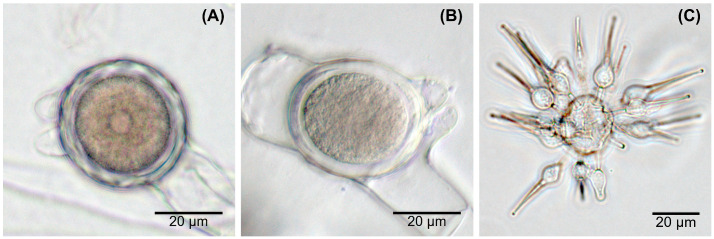
Zygospore wall undulation and exogenous microspores of *Basidiobolus* spp. (**A**) undulated zygospores from isolate E4; (**B**) smooth zygospore formed by isolate GP8; (**C**) microspores produced by *B. microsporus* DSM 3120.

The production of undulated, beaked zygospores (Figure 1A) could be observed in older, mature cultures of isolate E4 and both gecko fecal isolates collected in Clarens (Cla6, C3-1) as well as in the type strain *Basidiobolus microsporus* DSM 3120, while only smooth, beaked zygospores (Figure 1B) were produced by the eight other isolates. However, zygospores were not regularly produced, and two isolates (G10 and Ag5-5) lost the ability soon after isolation during sub-culturing. Exogenous microspores were only detected in older cultures of the type strain *Basidiobolus microsporus* (Figure 1C), even though not regularly. A whitish, aerial mycelium was sometimes formed by the isolates tested, most often and in larger amounts by isolates G9, GA7, Ag3, and GP8. However, such whitish mycelium was rarely observed for the reptile isolates G10, GA2, Ag5-5, and GP4. The production of odor (a distinct “streptomyces”-or “cellar”-like, sometimes more aromatic scent) occurred on a very irregular base.

Some strains tended to produce occasionally satellite colonies within the first days of incubation between 20 and 37 °C, but rarely at 40 °C. This resulted in a faster extension of the radial growth diameter as a ring of satellite colonies formed from conidia shot into the surroundings from the mother mycelium (Figure S1). However, at the lowest temperature tested (6 °C), the production of satellite colonies was never detected for any isolate. 

**Figure 2:**
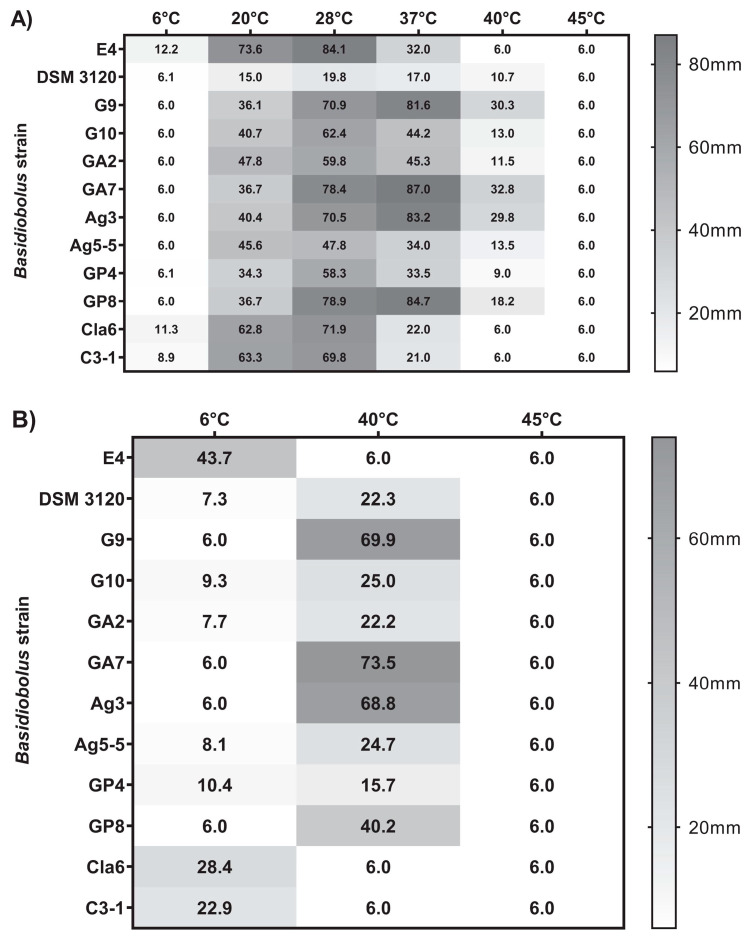
Growth temperature profiles of *Basidiobolus* reptile isolates and *B. microsporus* DSM 3120 based on average maximum colony diameter after (**A**) 4 days of incubation on Sabouraud Dextrose Agar (SDA) at six different temperatures between 6 and 45 °C and (**B**) 14 days of incubation on SDA at 6, 40, and 45 °C. Plugs of 6 mm diameter were used for inoculation.

The average maximum colony diameters recorded after 96 h (4 days) were used for comparing growth at 20, 28, and 37 °C, as some strains always reached the full Petri dish diameter within 7 days at these temperatures, while for 6, 40, and 45 °C, the maximum growth diameter after 14 days was used (Table S2). Generally, all strains tested could grow very well at 28 °C with the type strain showing the slowest, poorest growth (Figure 2A). For most strains, the radial growth decreased at 37 °C except for isolates G9, GA7, Ag3, and GP8, which grew faster at 37 °C than at 28 °C based on the growth diameter reached within 4 days (Figure 2A). These four isolates grew even quite well at 40 °C, reaching an average radial growth diameter of about 70 mm (isolates G9, GA7, Ag3) and about 40 mm (isolate GP8) within 14 days, while all other strains tested showed more restricted (isolates G10, GA2, Ag5-5, GP4, DSM 3120) or even no (isolates E4, Cla6, C3-1) growth within 14 days at 40 °C (Figure 2B). On the other hand, the three isolates E4, Cla6, and C3-1 grew at 6 °C within 4 days and reached an average diameter of about 25 to 44 mm within 14 days, while isolates G10, GA2, Ag5-5, GP4, and the type strain DSM 3120 showed only very restricted growth within 14 days (Figure 2B). Neither growth nor hyphae formation was detectable at 6 °C for isolates G9, GA7, Ag3, and GP8, and none of the 12 strains tested was able to grow at 45 °C within 14 days. Based on these data, three physiological groups could be differentiated in addition to the type strain *B. microsporus* DSM 3120: group I (E4, Cla6, C3-1) was able to grow at lower temperatures of 6 °C but not at 40 °C, group II (G10, GA2, Ag5-5, GP4) had an intermediate growth temperature profile, and group III (G9, GA7, Ag3, and less well GP8) was able to grow even at higher temperatures of 40 °C but not at 6 °C (Table 3). A cluster analysis confirmed this growth temperature-based grouping of isolates (Figure S2A). 

### 3.2. Sequence-Based Characterization

When using the genus-specific primer pair BasF611/BasR1340, all strains, including the type strain, showed the expected amplicon size of 730 bp. However, with the diagnostic group-specific primer pair Ba1/Ba2, no typical amplicon of 651 bp size was observed for the type strain *B. microsporus*, while all reptile feces isolates tested positive, confirming that none of the reptile isolates could be assigned to *B. microsporus*.

As these genus and group-specific primers cover partial regions of the 18S and 28S rRNA gene sequence, respectively, we analyzed whether the isolates showed sequence variations within these regions, which could be used for further strain differentiation. Alignment of the composite 18S rRNA sequence (1708 bp) using GenBank showed high sequence similarity (≥99%) for all tested strains to 13 deposited matching sequences of *Basidiobolus* strains (including four type strains and one uncultured clone) and one misidentified *Conidiobolus coronatus* strain (accession no. JQ014011.1). Such intrusion by one member of the genus *Conidiobolus* was reported previously in a fungal phylogeny study [50]. Therefore, a differentiation of the isolates at the species or subspecies level based on the 18S rRNA gene sequence was considered unreliable.

More sequence variability was observed within the sequences obtained for the D1/D2 domain of the 28S rRNA gene region, resulting in a consensus sequence of 738 bp for all reptile isolates and 737 bp for *B. microsporus* DSM 3120. As expected, analysis of these sequences using GenBank (BLAST) confirmed high similarity (99.2% and 100%) of the type strain *Basidiobolus microsporus* DSM 3120 used in this study with the two deposited sequences of *Basidiobolus microsporus* type material (CBS 130.62). Although one sequence of *B. magnus* type material (CBS 205.64; acc. no. JX242588.1) also showed a high similarity (99.5%) to *B. microsporus* DSM 3120, both of the other sequences deposited for the same type strain *B. magnus* (CBS 205.64 and ATCC 15379, acc. no. MH870046.1 and EF392425.1) had only 91.9% similarity, indicating that the identity of the first sequence of *B. magnus* (acc. no. JX242588.1) is questionable. Consequently, this single sequence was not considered for phylogenetic analysis. All the reptile isolates showed sequence similarities (BLAST) between 91.6 and 99.6% to the nine sequences available for type strains. However, no type strain material of *B. ranarum* is available. The highest similarities of over 99% were established for isolates G10, GA2, GP4, and Ag5-5 to two sequences of *B. heterosporus* (CBS 311.66 and ATCC 16580), with similarities <96.1% to all other *Basidiobolus* spp. type material sequences available. Isolates E4, Cla6, and C3-1 showed the highest similarities (>99% for E4 and >98.4% for Cla6 and C3-1) to two sequences of *B. magnus* type strains (CBS 205.64 and ATCC 15379), while similarities to all other *Basidiobolus* spp. type strain sequences were below 94.1% for these three isolates. The highest sequence similarities were obtained for isolates G9, GA7, and Ag3 to *B. meristosporus* (CBS 140.55, 98.8%) and *B. haptosporus* var. *minor* (ATCC 16579, 97.4%), while similarities to all other *Basidiobolus* spp. type strain sequences were <95.8%. The sequence obtained for isolate GP8 showed the highest similarity of 97.4% to the type strain *B. haptosporus* var. *minor* (ATCC 16579) and 97.2% similarity to *B. meristosporus* (CBS 140.55).

**Figure 3:**
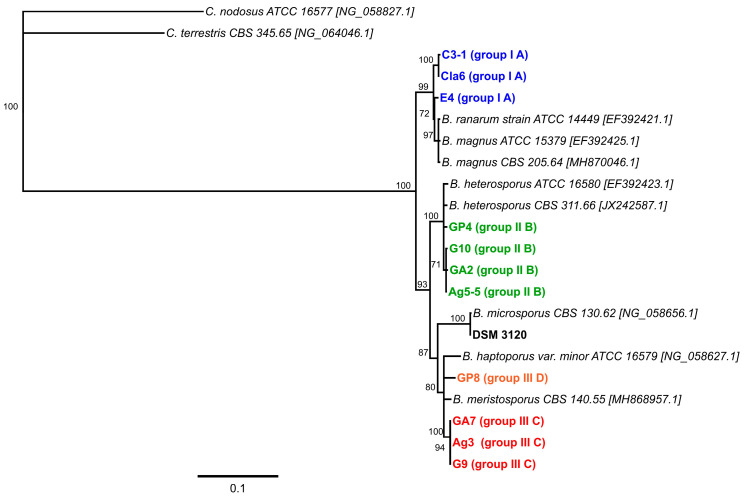
Phylogenetic tree constructed using neighbor-joining analysis (Geneious Prime Version 2020.2, 1000 bootstrap replicates) of the D1/D2 domain of the 28S rRNA gene sequences established in this study for the 11 *Basidiobolus* isolates from reptile feces and the type strain *Basidiobolus microsporus* DSM 3120. Sequences from two *Conidiobolus* type strains were used as an outgroup. Only support values of >70% are shown. The scale bar indicates substitutions per site. The color code matches the isolate characteristics specified in Table 3.

A phylogenetic tree established based on the partial 28S rRNA gene (D1/D2 domain) sequences revealed that the *Basidiobolus* isolates from reptile feces clustered into three distinct groups with *B. microsporus* forming a fourth cluster (Figure 3). 

Interestingly, the observed grouping of the isolates matches the clustering obtained by the analysis of their growth temperature profiles (Table 3, Figure S2). The corresponding distance matrix shows that all four established clusters share less than 96.2% sequence identity with each other (Table S3).

Further analysis of the 28S rRNA gene sequence revealed that for the type strain used, *Basidiobolus microsporus* DSM 3120, the reverse primer Ba2 does not have an appropriate binding site. This explains why *B. microsporus* does not produce the expected PCR amplicon when using the group-specific diagnostic primer pair Ba1/Ba2. For all other *Basidiobolus* reptile isolates, the sequence analysis of the 28S rRNA gene predicted an amplicon of 651bp length for the group-specific primer pair Ba1/Ba2 as previously described [41] and confirmed by PCR analysis in this study. Enzyme restriction patterns for this PCR amplicon sequence were predicted in silico, allowing for the differentiation of the reptile isolates. Three enzymes were chosen (HinP1I, HaeIII, and AccI), and restriction fragment length polymorphism (RFLP) analysis confirmed the predicted restriction patterns. Digestion of the group-specific PCR amplicon with HinP1I revealed three clusters of reptile isolates with identical restriction patterns (Figure S3): one group contained isolates E4, Cla6, and C3-1, one group contained isolates G9, GA7, and Ag3 and the third group remained uncut for isolates G10, GA2, Ag5-5, GP4, and GP8. Restriction analysis of the Ba1/Ba2 PCR product with HaeIII allowed no differentiation between the first two groups as established with HinP1I but highlighted that isolate GP8 had a unique restriction profile (Figure S4). The clearest differentiation was obtained using the restriction enzyme AccI (Figure S5), which resulted in the following clustering of reptile isolates: group A—isolates E4, Cla6, C3-1; group B—isolates G10, GA2, Ag5-5, GP4; group C—isolates G9, GA7, Ag3; group D—isolate GP8 (Table 3).

## 4. Discussion

The genus *Basidiobolus* is a long known and well-established taxonomic unit. Since the first report by Eidam in 1886, with *Basidiobolus ranarum* as the original type species isolated from frog excrements, two other species of this fungal genus were described from lizards and plant detritus [1,51,52]. However, both species, *B. lacertae* and *B. myxophilus*, became soon questionable and were considered identical with *B. ranarum* [26,51,53]. Unfortunately, no type cultures from any of these species were deposited when first described, as new isolates could be easily obtained from nature and, maybe, appropriate official culture collections were not yet established. Until the late 1960s, more *Basidiobolus* species had been described based on phenotypical differences such as the form of zygospores, aerial hyphae formation, the production of exogenous microspores, odor production during growth, as well as growth temperature preferences [2,3,4,22,23,24]. Today, five different type species (*B. haptosporus* var. *minor*, *B. heterosporus*, *B. magnus*, *B. meristosporus*, *B. microsporus*) and two non-type species (*B. ranarum* and *B. haptosporus*) are still available at well-known culture collections such as the ATCC and the CBS. However, in the meantime, a number of *Basidiobolus* strains were renamed to *B. ranarum*.

The production of undulated versus smooth zygospores is one of the major morphological characteristics traditionally used to divide the different *Basidiobolus* species, whereby the production of smooth zygospores was usually related to *Basidiobolus* spp. isolated from human or animal infections. Undulated zygospores are originally only attributed to *B. ranarum*, *B. magnus*, and *B. microsporus*, the latter additionally producing exogenous microspores [1,3,24]. The exclusive production of smooth zygospores occurs in *B. haptosporus* (including *B. haptosporus* var. *minor*) and *B. meristosporus* [3,4,22,23], while both types of zygospores apparently occur in *B. heterosporus* [4].

The occurrence of both rough and smooth zygospores in one culture at the same time rendered this feature a questionable species indicator [29]. However, as previously highlighted by several authors, zygospore wall characteristics are age-dependent; young zygospores always start with a smooth outer wall, and only the mature forms are undulated, which sometimes lose their undulation again shortly before germinating [1,3,5,24,25]. Therefore, as long as the culture is still alive and growing, both undulate and smooth states can co-occur. Additionally, as previously reported and also observed for some strains in this study, the production of zygospores depends on culture conditions, and isolates can lose their ability to produce zygospores during sub-culturing [9,11,23,29,30]. Therefore, although zygospores might be considered as a discrimination feature not necessarily reliable for species identification, the presence or absence of undulated zygospores in aged cultures (when produced) can nevertheless be used to separate strains. In this study, we were able to detect undulated zygospores only in three reptile isolates (E4, Cla6, and C3-1) and in the used type strain *Basidiobolus microsporus* DSM 3120, which was also the only one showing the formation of microspores. No other *Basidiobolus* species is reported to produce microspores, which is therefore considered as a reliable characteristic for identifying *B. microsporus* [3,25,29,30,31]. Therefore, none of the *Basidiobolus* isolates from reptiles analyzed in the present study belongs to the species *B. microsporus*.

The generally larger size of zygospores, conidia, and hyphae in *B. magnum* was primarily used for the differentiation of this species from *B. ranarum* [24], albeit otherwise, these two species are similar in their morphological characteristics. However, as dimensions of zygospores and hyphae can vary considerably in different isolates and strains depending on culture conditions [5,26,27,29], species differentiation on the base of size alone is doubtful. As the isolates analyzed in this study showed similar culture-dependent size variations of cellular features, this criterium was not used to differentiate isolates. 

Two characteristics historically used to differentiate the previously mentioned *Basidiobolus* species, the formation of aerial hyphae and distinctive odor production, were discussed as controversially as the previously mentioned size and undulation features. A distinctive musty, streptomyces-like odor (sometimes also reported as similar to benzene hexachloride) was first mentioned by Drechsler for cultures of *B. ranarum* and *B. magnus* [22,24], while no distinctive odor production has been described for any other *Basidiobolus* species. The production of aerial hyphae, on the other hand, was used for further distinguishing smooth zygospore producing species, with *B. haptosporus* showing no or only meager production of aerial mycelium while *B. meristosporus* produces well-developed whitish mycelium [3]. As many morpho-physiological criteria appeared to be very variable and affected by culture conditions or might even change during sub-culturing [6,13,27], they were later precluded as useful criteria for species differentiation [28,29]. Odor production was irregular and varied in the 11 reptile isolates of the current study from a faint to a distinctive cellar-like to sometimes more pungent aromatic smell, confirming these previously reported variabilities. The same applied to the formation of aerial hyphae, which was indicated by the presence of whitish mycelium in well-grown cultures. Hence, these two features, used originally for the differentiation of *Basidiobolus* species, were ruled out for separating the reptile isolates of this study. 

Determining temperature preferences for the differentiation between potentially pathogenic and non-pathogenic isolates is a reasonable approach, as a microorganism failing to grow at the human body temperature of about 37 °C is unlikely to cause human infection. This ability to grow better at 37 °C was previously used as an additional feature to differentiate *B. meristosporus* from *B. haptosporus*, suggesting that only *B. meristosporus* is a pathogenic species, which can grow even at 40 °C [25], while *B. haptosporus* is adapted to lower temperatures [23]. This adaptation to a lower temperature is supported by subsequent studies investigating growth temperature profiles for different *Basidiobolus* species, which indicate better growth for *B. haptosporus* reference strains at 25 °C than 37 °C [25,29]. However, at the same time, it was suggested that all pathogenic strains should be assigned to *B. haptosporus* as all human pathogenic isolates have smooth zygospores, while characteristics such as aerial hyphae and odor formation are unreliable for species identification [4,28]. As all isolates in one of these two studies were reported to grow at 24–28 °C as well as at 37 °C (unfortunately without clearly stating the optimum), the authors proposed the differentiation into several varieties, namely *B. haptosporus* var. *haptosporus* (for the original *B. haptosporus*), B. *haptosporus* var. *meristosporus* (for *B. meristosporus*), and *B. haptosporus* var. *minor* (as a new variety) [4]. Subsequently, only *B. haptosporus* was assumed to be pathogenic and mainly reported as a causative agent for infections [4,7,9,15,28,30]. This contributed further to the taxonomic dilemma within the genus *Basidiobolus*. 

The ability of *Basidiobolus* spp. isolates from humans to grow better at 37 °C is regularly reported and expected on microbiological grounds [25,29,54]. However, several studies pointed out that although environmental isolates often showed an optimum growth at about 25 °C, many were still able to grow at 37 °C, while not all non-human isolates failed to grow better at the higher temperature [7,9,25,29,54,55,56]. Yet, this is not a contradiction, as non-human sources can be the origin of potentially pathogenic strains. Still, distinguishing *Basidiobolus* species based on growth temperature preferences was no longer followed, and until now, only *Basidiobolus ranarum* is considered to be pathogenic. 

All 12 strains examined in the present study were able to grow at 37 °C, albeit to varying degrees, indicating that all 12, including *B. microsporus*, could be potentially pathogenic. However, as pathogenicity does not only depend on the ability to grow at 37 °C, further studies need to investigate which additional factors govern the pathogenicity of *Basidiobolus* spp. 

A few studies additionally analyzed the ability of human and environmental isolates to grow at higher temperatures (≥40 °C) and/or at low temperatures (≤15 °C). Interestingly, while all clinical isolates (human and animal) tested in different studies showed growth (although reduced) at 40 °C [25,55], no growth was reported for any clinical isolates tested at low temperatures [5,54,55]. On the other hand, environmental isolates were reported to grow at temperatures of 15 °C or even 7 °C [5,54,55]. However, restricted growth at 40 °C was also reported for environmental isolates [7,11,25,55,56]. These studies indicate that pathogenic isolates potentially differ from environmental isolates less likely to cause infections, especially regarding their minimum growth temperature.

The temperature preferences for the growth of isolates proved to be a useful criterium to separate the reptile isolates of this study into three distinct groups, one group of isolates able to grow even at temperatures below 10 °C, one group growing best at ambient but very restricted at lower and higher temperatures, and the last group growing best at 37 °C and even growing quite well at 40 °C. 

Interestingly, these physiological clusters matched the grouping of the reptile isolates obtained via phylogenetic analysis in the present study. Although the 18S rRNA gene sequence analysis did not support a differentiation at the species level (sequence similarities >99%), the 28S rRNA gene D1/D2 domain sequence analysis enabled the potential assignment of isolates to type species originally described based on physiological and morphological characteristics. A problem arises from the inconsistent use of species names within the genus *Basidiobolus*, as the species name assigned to pathogenic isolates in medically related studies changed over time from *B. ranarum* to *B. meristosporus* and *B. haptosporus* and finally back to *B. ranarum*, with different species names frequently used in the literature for the description of environmental and clinical *Basidiobolus* isolates. Therefore, sequences deposited in GenBank might be incorrectly annotated due to such taxonomic changes [40,50]. Additionally, sequence data quality depends on sequencing accuracy (errors) and might be further compromised by PCR-based errors, culture contamination, or mislabeling of original materials [57,58]. All this might lead to fungal misidentification, and exclusively sequence-based identification of *Basidiobolus* species should be applied cautiously. To minimize such problems, only sequences from type material or certified reference cultures (for *B. ranarum*) were used for phylogenetic analysis in this study. 

Based on the results from this study, all three isolates able to grow at low temperatures (E4, Cla6, C3-1) showed the highest similarity to *Basidiobolus magnus* and *B. ranarum*. This is in line with the characteristics originally described for these species that are the formation of undulated zygospores and preference for lower temperatures [1,3,24,25]. The group with optimum growth at higher temperatures clustered with *Basidiobolus meristosporus*, previously described to grow better at 37 °C [25], and *B. haptosporus* var. *minor*, which appeared as a subcluster in this group together with the isolate GP8. Interestingly, isolate GP8 was growing slightly less well at 40 °C than the other three reptile isolates G9, GA7, and Ag3 of this group. Unfortunately, more detailed physiological studies seem not to be available for *B. haptosporus* var. *minor*, and the original description of this variety does not explicitly mention any temperature preferences [4]. The third cluster of isolates (G10, GA2, Ag5-5, GP4) showed the highest similarity to type strain material of *B. heterosporus*, which was reported to grow better at 25 °C than at 37 °C [29]. The reptile isolates clustering in this group grew better at 28 °C than at 37 °C, suggesting a similar growth temperature preference. However, in contrast to the morphological characteristics initially described for *B. heterosporus* [4], only smooth zygospores were formed by the reptile isolates before two isolates lost the ability to produce zygospores. This underlines the challenges of using only morphological features for the reliable differentiation of isolates in the genus *Basidiobolus*.

No threshold value is currently defined for fungal species determination based on 28S rRNA gene sequences similarity. Therefore, sequence differences in the D1/D2 domain of 28S rRNA established in the current study for *Basidiobolus* isolates cannot be directly employed for species identification. However, assuming a 97% similarity as cutoff value for species identification suggested and applied for the fungal ITS region [40], the four clusters revealed in our study might represent at least four different species. Among these, *B. microsporus* is clearly distinct from all other species, which is in line with previous findings [3,25,29,31,32,33,34]. Similarly, *B. heterosporus* seems to form a distinct cluster, while *B. ranarum* cannot be distinguished from *B. magnus* and should therefore be considered as one species. Finally, *B. meristosporus* and *B. haptosporus* var. *minor* can be distinguished from the other three clusters containing *B. microsporus*, *B. heterosporus* and *B. ranarum*, respectively. Yet, as the type strains of *B. meristosporus* and *B. haptosporus* var. *minor* exhibit slightly less than 97% sequence identity based on the phylogenetic analysis performed in this study, they might be not considered as separate species but as varieties of one species (*B. haptosporus*), as suggested in the past [4]. The presence of four clusters matches a recent taxonomic study on the basal clades of fungi, proposing four species within the genus of *Basidiobolus* [59].

Although the reptile isolates could be assigned to specific *Basidiobolus* spp. type strains, it is still possible that the sequence differences within parts of the 28S rRNA gene are due to genetic variation within a species. Previous studies suggested the presence of large genetical heterogeneity in the genus *Basidiobolus* with only *B. microsporus* being clearly distinct, but at the same time, they indicated that isolates differ to a certain degree by their origin (clinical/non-clinical and geographic) [33,34]. Additionally, *Basidiobolus meristosporus* was estimated to possess large copy numbers—exceeding 1000—for ribosomal RNA genes [60]. In turn, this might result in a higher frequency of sequence variations being present in a strain, allowing strains to adapt faster to changes in growth conditions and explaining the large physiological plasticity and strain variability observed in this genus [13,26,29]. 

The results from the current study show that the morpho-physiological characterization of the reptile isolates matches the grouping obtained using 28S rRNA (D1/D2 domain) gene sequence analysis. As cultural methods are time-consuming, the combination of a PCR-based identification followed by RFLP analysis seems to be a reliable approach for a faster differentiation of *Basidiobolus* isolates. For the genus level confirmation of *Basidiobolus*, the 18S rRNA based primer pair should be used, while the 28S rRNA targeting primer pair Ba1/Ba2 proved to exclude *Basidiobolus microsporus*, as this strain lacks the binding site for the second primer Ba2. For isolate differentiation, the best result was obtained digesting the diagnostic PCR amplicon Ba1/Ba2 with AccI, which allowed distinguishing all groups identified in this study, including the subcluster of isolate GP8.

Even though basidiobolomycosis is a rare disease, it often affects healthy humans and can manifest as severe and difficult to diagnose infection [18,19,20,21,35,44]. From a diagnostic point of view, it might be sufficient to identify *Basidiobolus* isolates at the genus level as the cause of an infection. However, to better understand possible infection routes and estimate potential health risks associated with the presence of *Basidiobolus* spp. in the environment, it is vital to confirm the type of strain causing an infection and its origin. Comparing RFLP patterns from clinical and environmental isolates might allow source tracking. Although the culture-based examination will still be necessary to characterize isolates properly, RFLP analysis is a fast screening method that can identify groups of similar isolates from which selected representatives can be further analyzed. The ability of restriction analysis of ribosomal RNA gene sequences to differentiate between human and saprobic *Basidiobolus* isolates was previously shown [34]. Unfortunately, we had no access to clinical *Basidiobolus* spp. isolates for direct comparison of clinical and environmental isolates. However, in silico AccI restriction analysis of the diagnostic Ba1/Ba2 amplicon extracted from selected sequences deposited in GenBank for *Basidiobolus* spp. strains of human origin resulted in RFLP patterns similar to those established in this study using AccI. Albeit the RFLP pattern showed some variation, most human isolates tested in silico apparently possess an RFLP pattern matching the one from the higher temperature adapted isolates G9, Ag3, and GA7 or isolate GP8 (Figure S6), suggesting that not all environmental isolates are pathogenic. However, these data should be confirmed in situ, if possible, with both reference strains from culture collections as well as clinical isolates. 

## 5. Conclusions

This study confirms that morpho-physiological characteristics such as zygospore wall undulation, odor formation, or production of whitish mycelia are highly variable features that do not allow for a reliable differentiation of members of the genus *Basidiobolus*. Similarly, the 18S rRNA gene sequence proved to be unsuitable for distinguishing environmental isolates. However, the separation of isolates into clusters based on growth temperature preference and RFLP analysis of a diagnostic PCR amplicon (located on the 28S rRNA gene) was possible and supported by the phylogenetic comparison of the 28S rRNA gene (D1/D2 domain) sequence. In silico RFLP analysis of the diagnostic PCR amplicon sequence of selected clinical isolates suggests that pathogenic strains mainly fall into the cluster of higher temperature adapted members of the genus *Basidiobolus*. 

Additional work combining physiological studies along with multi-gene and whole-genome analyses is required to elucidate the *Basidiobolus* species/strain complex, reveal possible cryptic species, and improve our understanding of factors governing strain pathogenicity. Therefore, the comparison of certified type and reference strains with environmental and clinical isolates from different geographical regions would be essential to clarify unresolved issues for this fascinating fungal genus. 

Still, the RFLP analysis suggested in our study would be a useful approach for epidemiological studies.

## Figures and Tables

**Table 1:** Origin of *Basidiobolus* isolates used in the present study and GenBank accession numbers of the submitted 18S rRNA (SSU) and partial 28S rRNA (LSU, D1/D2 domain) gene sequences.

Isolate	GenBank Accession Numbers	Origin/Country	Source
Ag3	SSU: MW133784	Outside (garden) Pietermaritzburg, South Africa	Agama feces
	LSU: MW135353		
Ag5-5	SSU: MW135344		
	LSU: MW135352		
GP4	SSU: MW135349		
	LSU: MW135331		
GP8	SSU: MW130090		
	LSU: MW135345		
E4	SSU: MW135334	Inside (house) Pietermaritzburg, South Africa	Gecko feces
	LSU: MW135343		
G9	SSU: MW135351		
	LSU: MW135329		
G10	SSU: MW135341		
	LSU: MW135335		
GA2	SSU: MW127177	Outside (house) Pietermaritzburg, South Africa	
	LSU: MW135038		
GA7	SSU: MW127174		
	LSU: MW135350		
Cla6	SSU: MW127175	Outside (house) Clarens, South Africa	
	LSU: MW135307		
C3-1	SSU: MW135346		
	LSU: MW135305		
Bm DSM3120	SSU: MW127176	Culture collection DSMZ, Germany	Type strain
	LSU: MW135342		

**Table 2:** Primer and PCR conditions used for the amplification of various parts of 18S rRNA and partial 28S rRNA gene (D1/D2) sequences.

	Primer Pair	Reference	Target	Approximate Size	35 Cycles of
1	BasF611 BasR1340	[43]	*Basidiobolus* genus specific (18S rRNA)	≈730 Bp	30 s at 94 °C 45 s at 62 °C 60 s at 72 °C
2	Ba1 Ba2	[41]	*B. ranarum* group specific (28S rRNA)	≈651 bp	
3	FF1 FR1	[45]	Fungi specific (18S rRNA)	≈633 bp	30 s at 94 °C 30 s at 50 °C 60 s at 72 °C
4	NS1 NS4	[46]	Partial 18S rRNA region	≈1138 bp	
5	NS5	[46]	Partial 18S rRNA region	≈662 bp	30 s at 94 °C 30 s at 55 °C 45 s at 72 °C
	NS8Z	[47]			
6	NL1 NL4	[48]	Partial 28S rRNA region (D1/D2 domain)	≈781 bp	30 s at 94 °C 30 s at 55 °C 60 s at 72 °C

**Table 3:** Summary for zygospore formation, growth temperature profile, Ba1/Ba2 PCR product, AccI RFLP pattern, and highest 28S rRNA (D1/D2 domain) sequence similarity (BLAST, type strain sequences) for *Basidiobolus* reptile isolates and *B. microsporus* DSM 3120.

Isolate	Highest Sequence Similarity (%; Accession Number)	Zygospore Formation	Group by Growth Temperature	Ba1/Ba2 PCR (651 bp)	Group by AccI RFLP (Fragment Sizes in bp)
**E4**	*B. magnus* ATCC 15379 (99.2%; EF392425.1)	undulate	**I** (lower temp, no growth at 40 °C)	Positive	**A** (651)
**Cla6**	*B. magnus* CBS 205.64 (98.8%; MH870046.1)	undulate		Positive	
**C3-1**	*B. magnus* CBS 205.64 (98.6%; MH870046.1)	undulate		Positive	
**G10**	*B. heterosporus* CBS 311.66 (99.5%; JX242587.1)	smooth (lost)	**II** (intermediate)	Positive	**B** (122 + 137 + 392)
**GA2**	*B. heterosporus* CBS 311.66 (99.5%; JX242587.1)	smooth		Positive	
**Ag5-5**	*B. heterosporus* CBS 311.66 (99.6%; JX242587.1)	smooth (lost)		Positive	
**GP4**	*B. heterosporus* ATCC 16580 (99.6%; EF392423.1)	smooth		Positive	
**G9**	*B. meristosporus* CBS 140.55 (98.8%; MH868957.1)	smooth	**III** (higher temp, no growth at 6 °C)	Positive	**C** (85 + 137 + 429)
**GA7**	*B. meristosporus* CBS 140.55 (98.8%; MH868957.1)	smooth		Positive	
**Ag3**	*B. meristosporus* CBS 140.55 (98.8%; MH868957.1)	smooth		Positive	
**GP8**	*B. haptosporus* var. *minor* ATCC16579 (97.4%; NG_058627.1)	smooth		Positive	**D** (137 + 514)
**DSM 3120**	*B. microsporus* CBS 130.62 (100%; NG_058656.1)	undulate	generally slow growth	Negative	not applicable

## Data Availability

Data are contained within this article and supplementary material. Sequence data are available via GenBank.

## Supplementary Materials

The following are available online at https://www.mdpi.com/2309-608X/7/2/110/s1, Table S1: Sequences of primers used in this study. Table S2: Average maximum growth diameter on Sabouraud Dextrose Agar (SDA) of 11 *Basidiobolus* isolates from reptile feces and of the type strain *Basidiobolus microsporus* DSM 3120 after incubation at 6, 20, 28, 37, 40, and 45 °C. Table S3: Distance matrix (% identity) based on 28S rRNA gene (D1/D2 domain) sequence comparison of *Basidiobolus* and *Conidiobolus* strains used for the phylogenetic analysis. Figure S1: *Basidiobolus* sp. isolate GA2 after growth at 20 °C for 7 days on Sabouraud Dextrose Agar. Figure S2: Clustering of *Basidiobolus* spp. isolates from reptile feces and *Basidiobolus microsporus* DSM 3120: (A) Dendrogram based on growth temperature profile after 4 days created with PAST 4.01 (www.nhm.uio.no) using UPGMA and Euclidian similarity index and (B) Cladogram based on partial 28S rRNA gene sequence (D1/D2 domain) created with Geneious Prime Version 2020.2 using UPGMA consensus analysis (1000 bootstraps). Figure S3: Agarose gel of HinP1I RFLP pattern after restriction of Ba1/Ba2 PCR product from reptilian *Basidiobolus* spp. isolates. Figure S4: Agarose gel of HaeIII RFLP pattern after restriction of Ba1/Ba2 PCR product from reptilian *Basidiobolus* spp. isolates. Figure S5: Agarose gel of AccI RFLP pattern after restriction of Ba1/Ba2 PCR product from reptilian *Basidiobolus* spp. isolates. Figure S6: AccI in silico restriction pattern of eight sequences from human isolates and four representative *Basidiobolus* spp. isolates (GP8, Ag3, G10, E4) covering all four restriction patterns established in this study (all trimmed to the Ba1/Ba2 amplicon size, created with Geneious Prime Version 2020.2).
