# Supplementary material for: Differentiation of Basidiobolus spp. Isolates: RFLP of a Diagnostic PCR Amplicon Matches Sequence-Based Classification and Growth Temperature Preferences
Source: J Fungi (Basel). 2021 Feb 3;7(2):110. doi: 10.3390/jof7020110 (PMC7913143; doi:10.3390/jof7020110)
Supplement: Supplementary file 1 [file jof-07-00110-s001.pdf]

Supporting information (Differentiation of *Basidiobolus* isolates, Claussen & Schmidt)

Table S1

Sequences of primers used in this study.

	Primer name	Sequence	Reference
1	BasF611	5'-CCAGACGGTCTGCCTGTTT-3'	[43]
	BasR1340	5'-TGTCCGTCTACACAAAGGTAAACG-3'	
2	Ba1	5'-AAAATCTGTAAGGTTCAACCTTG-3'	[41]
	Ba2	5'-TGCAGGAGAAGTACATCCGC-3'	
3	FF1	5′-GTTAAAAAGCTCGTAGTTGAAC-3′	[45]
	FR1	5′-CTCTCAATCTGTCAATCCTTATT-3′	
4	NS1	5'-GTAGTCATATGCTTGTCTC-3'	[46]
	NS4	5'-CTTCCGTCAATTCCTTTAAG-3'	
5	NS5	5'-AACTTAAAGGAATTGACGGAAG-3'	[46]
	NS8Z	5'-TCCGCAGGTTCACCTACG-3'	[47]
6	NL1	5'-GCATATCAATAAGCGGAGGAAAAG-3'	[48]
	NL4	5'-GGTCCGTGTTTCAAGACGG-3'	

Table S2

Average maximum growth diameter on Sabouraud Dextrose Agar (SDA) of 11 *Basidiobolus* isolates from reptile feces and of the type strain *Basidiobolus microsporus* DSM 3120 after incubation at 6, 20, 28, 37, 40 and 45°C.

**Isolate**	**Time**	**Average maximum growth diameter ± standard error (in mm) after incubation on SDA at**					
		**6°C**	**20°C**	**28°C**	**37°C**	**40°C**	**45°C**
**E4**	4d	12.2±0.31	73.6±0.69	84.1±2.73	32.0±1.06	ng	ng
	14d	43.7±3.02	na	na	na	ng	ng
**DSM 3120**	4d	6.1±0.13	15.0±0.58	19.8±0.82	17.0±0.97	10.7±0.61	ng
	14d	7.3±0.31	na	na	na	22.3±1.61	ng
**G9**	4d	ng	36.1±1.63	70.9±1.63	81.6±1.15	30.3±0.42	ng
	14d	ng	na	na	na	69.9±1.06	ng
**G10**	4d	ng	40.7±0.73	62.4±2.27	44.2±2.89	13.0±0.93	ng
	14d	9.3±0.73	na	na	na	25.0±2.83	ng
**GA2**	4d	ng	47.8±6.04	59.8±4.54	45.3±6.25	11.5±0.56	ng
	14d	7.7±0.36	na	na	na	22.2±1.05	ng
**GA7**	4d	ng	36.7±2.39	78.4±2.79	87.0±0.00†	32.8±1.99	ng
	14d	ng	na	na	na	73.5±1.34	ng
**Ag3**	4d	ng	40.4±1.89	70.5±2.46	83.2±1.72	29.8±2.86	ng
	14d	ng	na	na	na	68.8±4.57	ng
**Ag5-5**	4d	ng	45.6±1.03	47.8±0.70	34.0±1.15	13.5±0.85	ng
	14d	8.1±0.46	na	na	na	24.7±2.76	ng
**GP4**	4d	6.1±0.14	34.3±3.02	58.3±1.84	33.5±0.56	9.0±0.26	ng
	14d	10.4±0.95	na	na	na	15.7±2.16	ng
**GP8**	4d	ng	36.7±1.41	78.9±0.99	84.7±1.56	18.2±1.78	ng
	14d	ng	na	na	na	40.2±2.70	ng
**Cla6**	4d	11.3±0.87	62.8±1.42	71.9±0.90	22.0±1.53	ng	ng
	14d	28.4±2.16	na	na	na	ng	ng
**C3-1**	4d	8.9±0.55	63.3±0.75	69.8±0.36	21.0±0.27	ng	ng
	14d	22.9±1.33	na	na	na	ng	ng

Inoculation plugs of 6mm diameter were used from actively growing mycelia of isolates tested. All values were established from at least 6 measurements from 3 independent experiments.
ng – no growth detected
na – not applicable
† isolate always reached the full plate diameter of 87.0 mm

Table S3

Distance matrix (% identity) based on 28S rRNA gene (D1/D2 domain) sequence comparison of *Basidiobolus* and *Conidiobolus* strains used for the phylogenetic analysis.

Temp group	I							slow			II					III						
RFLP group	A							N/A			B					D	C					
	C3-1	Cla6	E4	*B.ran* ATCC	*B.ma* ATCC	*B.ma* CBS	*B.mi* CBS	DSM 3120	*B.het* ATCC	*B.het* CBS	GP4	GA2	Ag5-5	G10	*B.ha* ATCC	GP8	Ag3	G9	GA7	*B.me* CBS	*C.no* ATCC	*C.ter* CBS
C3-1		99.9	98.6	98.5	98.5	98.6	91.3	91.5	93.2	93.3	93.5	93.4	93.5	93.4	92.9	93.1	93.1	93.1	93.1	93.2	47.5	49.5
Cla6	99.9		98.8	98.6	98.6	98.8	91.1	91.3	93.3	93.4	93.6	93.5	93.6	93.5	92.7	93	93.2	93.2	93.2	93.4	47.7	49.7
E4	98.6	98.8		99.3	99.2	99.1	91.4	91.6	93.6	93.7	93.9	93.8	93.9	93.8	92.6	93.2	93.5	93.5	93.5	93.4	47.4	49.2
B. ranarum ATCC 14449	98.5	98.6	99.3		99.9	99.7	91.2	91.2	93.4	93.4	93.6	93.4	93.6	93.4	92.6	93	93.3	93.3	93.3	93.4	47.1	49.3
B. magnus ATCC 15379	98.5	98.6	99.2	99.9		99.9	91.1	91.2	93.4	93.4	93.5	93.4	93.5	93.4	92.6	93	93.3	93.3	93.3	93.4	47.4	49.4
B. magnus CBS 205.64	98.6	98.8	99.1	99.7	99.9		91	91.2	93.3	93.4	93.6	93.5	93.6	93.5	92.7	93	93.2	93.2	93.2	93.4	47.3	49.5
B. microsporus CBS 130.62	91.3	91.1	91.4	91.2	91.1	91		100	93.3	93.1	93.5	93.1	93.2	93.1	94	93.5	94.4	94.4	94.4	94.7	46.7	48.1
DSM 3120	91.5	91.3	91.6	91.2	91.2	91.2	100		93.3	93.1	93.6	93.2	93.4	93.2	94.1	93.6	94.6	94.6	94.6	94.9	46.5	48.1
B. heterosporus ATCC 16580	93.2	93.3	93.6	93.4	93.4	93.3	93.3	93.3		99.2	99.5	99	99.2	99.2	94.2	94.9	95.6	95.6	95.6	95.8	47.5	49.1
B. heterosporus CBS 311.66	93.3	93.4	93.7	93.4	93.4	93.4	93.1	93.1	99.2		99.3	99.5	99.6	99.5	94.2	95.2	95.7	95.7	95.7	95.7	47.3	49.4
GP4	93.5	93.6	93.9	93.6	93.5	93.6	93.5	93.6	99.5	99.3		99.6	99.7	99.6	94.5	95.1	95.9	95.9	95.9	95.9	47	49.1
GA2	93.4	93.5	93.8	93.4	93.4	93.5	93.1	93.2	99	99.5	99.6		99.9	99.7	94.6	95.3	95.8	95.8	95.8	95.8	47	49.2
Ag5-5	93.5	93.6	93.9	93.6	93.5	93.6	93.2	93.4	99.2	99.6	99.7	99.9		99.9	94.5	95.4	95.9	95.9	95.9	95.9	47.1	49.2
G10	93.4	93.5	93.8	93.4	93.4	93.5	93.1	93.2	99.2	99.5	99.6	99.7	99.9		94.4	95.5	95.8	95.8	95.8	96.1	47.1	49.2
B. haptoporus var. minor ATCC 16579	92.9	92.7	92.6	92.6	92.6	92.7	94	94.1	94.2	94.2	94.5	94.6	94.5	94.4		97.4	97.4	97.4	97.4	96.8	46.6	49.2
GP8	93.1	93	93.2	93	93	93	93.5	93.6	94.9	95.2	95.1	95.3	95.4	95.5	97.4		98.1	98.1	98.1	97.2	47.3	49
Ag3	93.1	93.2	93.5	93.3	93.3	93.2	94.4	94.6	95.6	95.7	95.9	95.8	95.9	95.8	97.4	98.1		100	100	98.8	46.7	48.8
G9	93.1	93.2	93.5	93.3	93.3	93.2	94.4	94.6	95.6	95.7	95.9	95.8	95.9	95.8	97.4	98.1	100		100	98.8	46.7	48.8
GA7	93.1	93.2	93.5	93.3	93.3	93.2	94.4	94.6	95.6	95.7	95.9	95.8	95.9	95.8	97.4	98.1	100	100		98.8	46.7	48.8
B. meristosporus CBS 140.55	93.2	93.4	93.4	93.4	93.4	93.4	94.7	94.9	95.8	95.7	95.9	95.8	95.9	96.1	96.8	97.2	98.8	98.8	98.8		46.3	48.8
C. nodosus ATCC 16577	47.5	47.7	47.4	47.1	47.4	47.3	46.7	46.5	47.5	47.3	47	47	47.1	47.1	46.6	47.3	46.7	46.7	46.7	46.3		68.1
C. terrestris CBS 345.65	49.5	49.7	49.2	49.3	49.4	49.5	48.1	48.1	49.1	49.4	49.1	49.2	49.2	49.2	49.2	49	48.8	48.8	48.8	48.8	68.1	

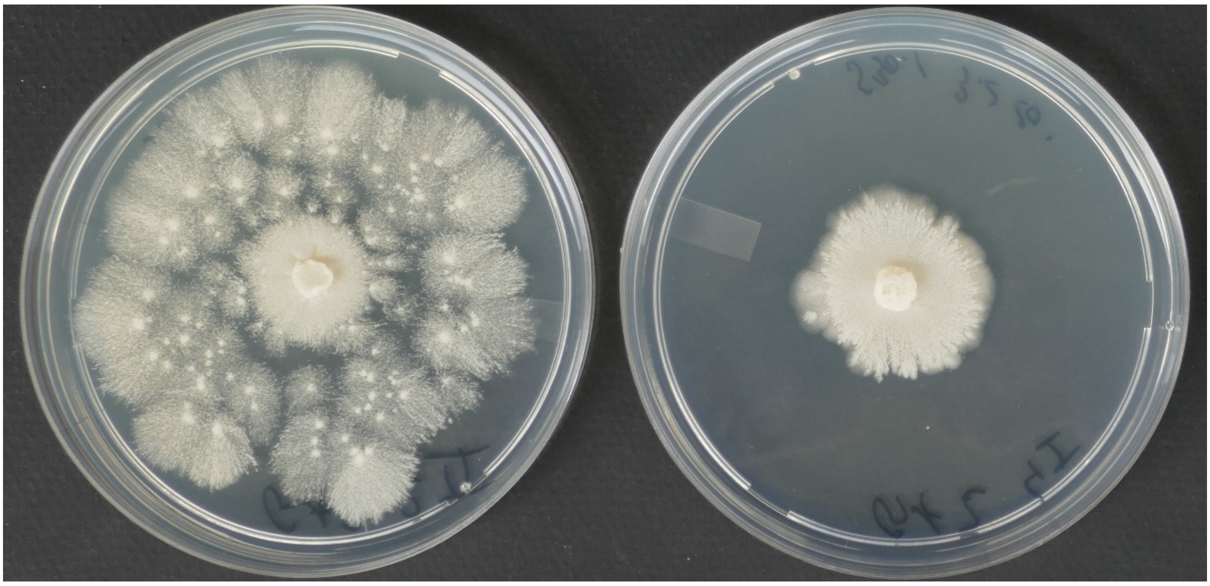

Figure S1

Basidiobolus sp. isolate GA2 after growth at 20°C for 7 days on Sabouraud Dextrose Agar. Left-hand plate with the production of satellite colonies due to conidia shot into the surrounding and right-hand plate without shooting.

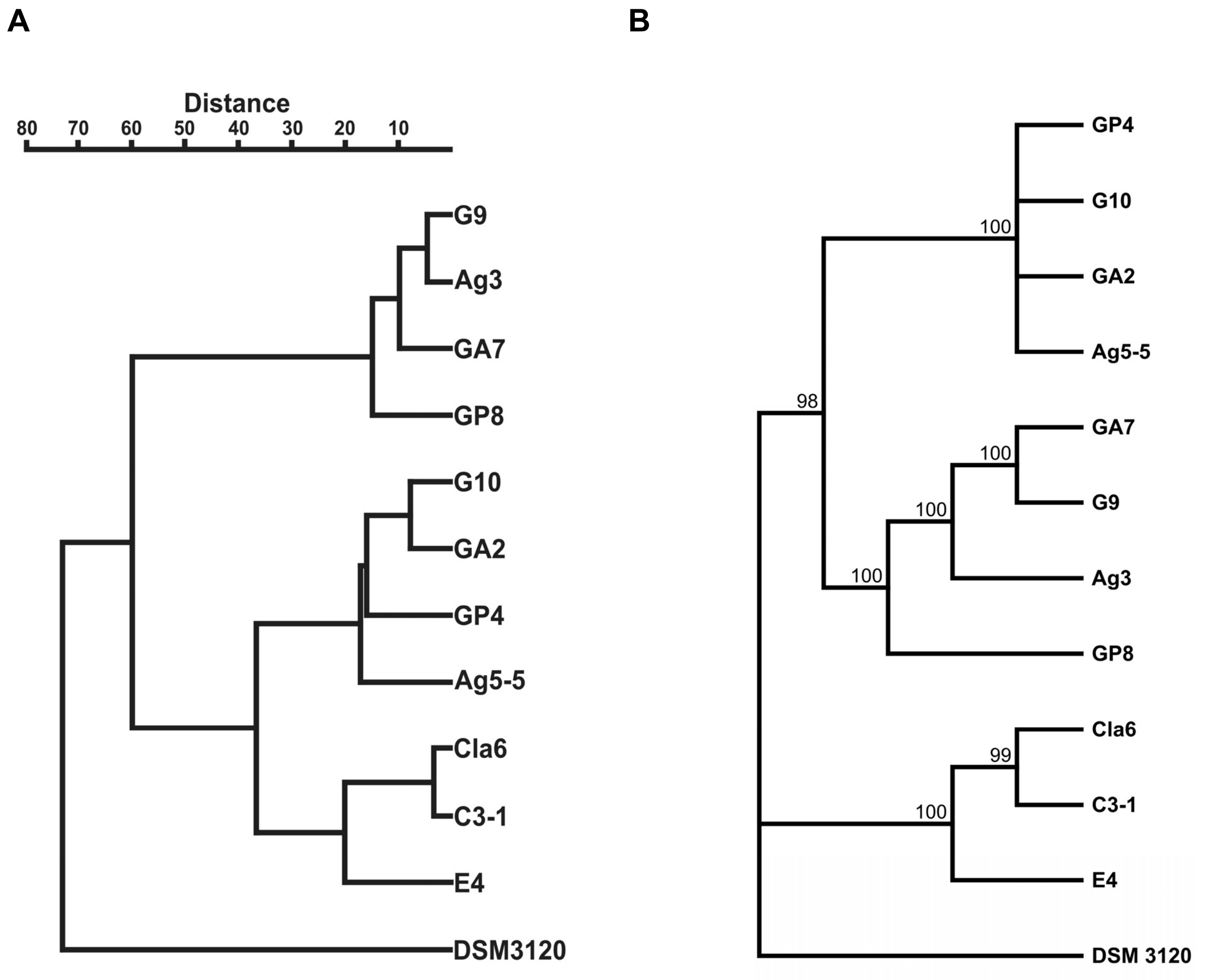

Figure S2

Clustering of *Basidiobolus* spp. isolates from reptile feces and *Basidiobolus microsporus* DSM 3120. **A**) Dendrogram based on growth temperature profile after 4 days created with PAST 4.01 (www.nhm.uio.no) using UPGMA and Euclidian similarity index and **B**) Cladogram based on partial 28S rRNA gene sequence (D1/D2 domain) created with Geneious Prime Version 2020.2 using UPGMA consensus analysis (1000 bootstraps).

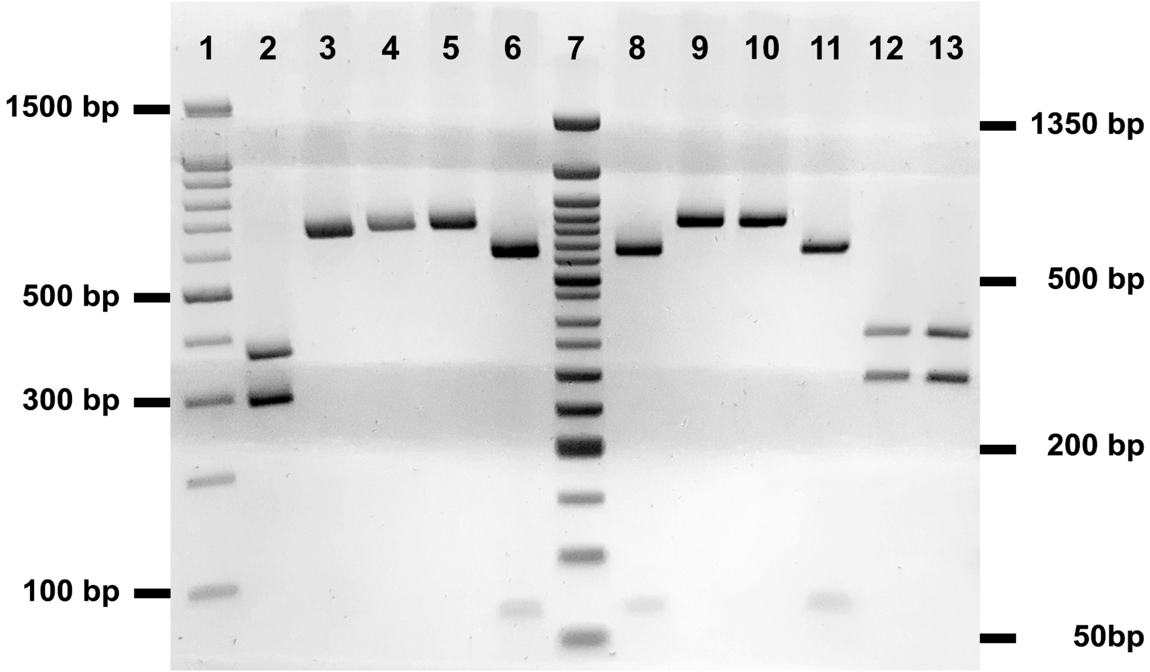

Figure S3

Agarose gel of HinP1I RFLP pattern after restriction of Ba1/Ba2 PCR product from reptilian *Basidiobolus* spp. isolates. Lane 2 – Cla6 (gecko); lane 3 – GP8 (agama); lane 4 – GP4 (agama); lane 5 – Ag5-5 (agama); lane 6 – Ag3 (agama); lane 8 – GA7 (gecko); lane 9 – GA2 (gecko); lane 10 – G10 (gecko); lane 11 – G9 (gecko); lane 12 – E4 (gecko); lane 13 – C3-1 (gecko). Molecular weight markers are placed in lane 1 (GeneDirex 100bp ladder, sizes of selected bands are indicated on the left hand) and in line 7 (NEB 50bp ladder, sizes of selected bands are indicated on the right hand).

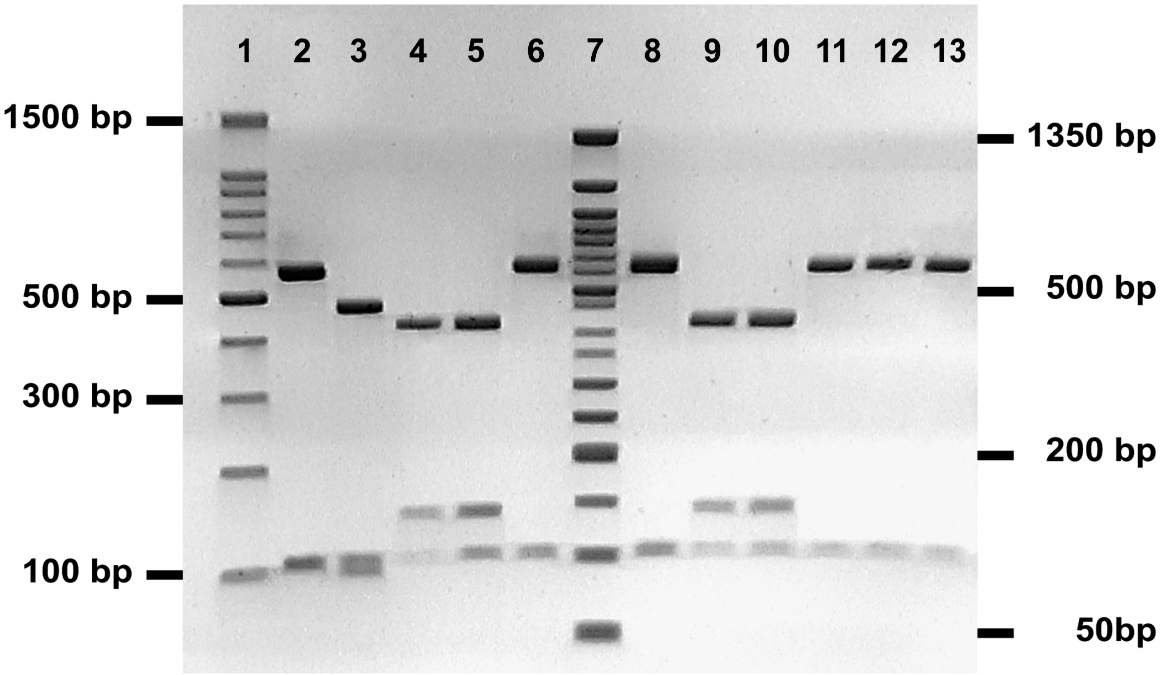

Figure S4

Agarose gel of HaeIII RFLP pattern after restriction of Ba1/Ba2 PCR product from reptilian *Basidiobolus* spp. isolates. Lane 2 – Cla6 (gecko); lane 3 – GP8 (agama); lane 4 – GP4 (agama); lane 5 – Ag5-5 (agama); lane 6 – Ag3 (agama); lane 8 – GA7 (gecko); lane 9 – GA2 (gecko); lane 10 – G10 (gecko); lane 11 – G9 (gecko); lane 12 – E4 (gecko); lane 13 – C3-1 (gecko). Molecular weight markers are placed in lane 1 (GeneDirex 100bp ladder, sizes of selected bands are indicated on the left hand) and in line 7 (NEB 50bp ladder, sizes of selected bands are indicated on the right hand).

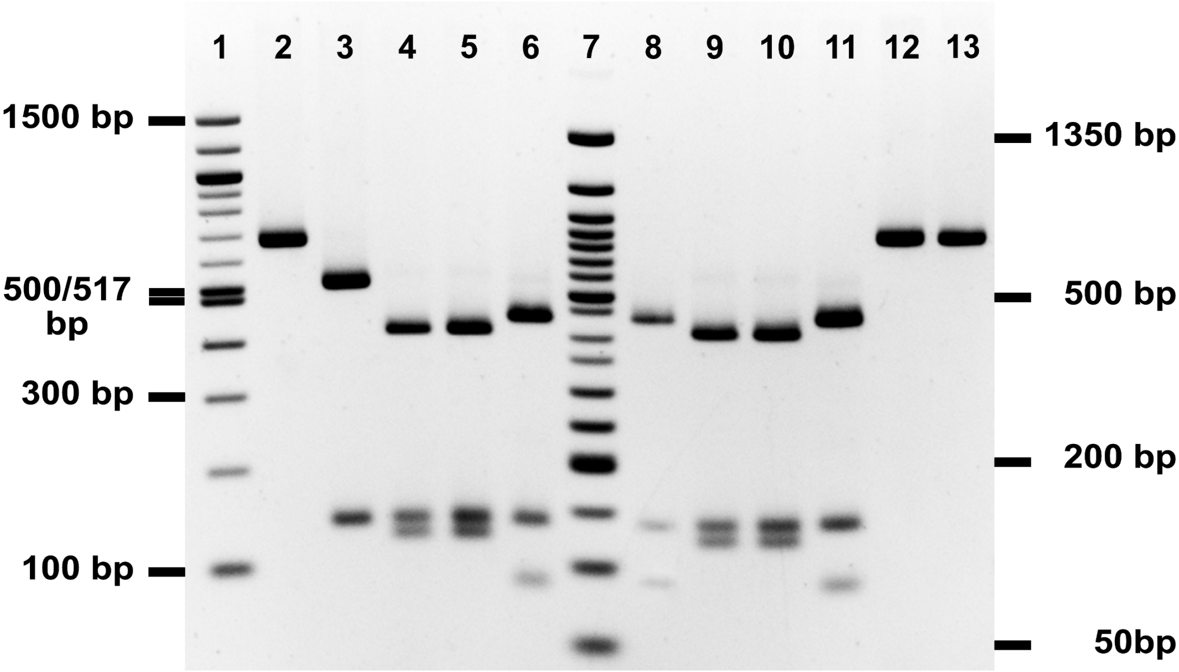

Figure S5

Agarose gel of AccI RFLP pattern after restriction of Ba1/Ba2 PCR product from reptilian *Basidiobolus* spp. isolates. Lane 2 – Cla6 (gecko); lane 3 – GP8 (agama); lane 4 – GP4 (agama); lane 5 – Ag5-5 (agama); lane 6 – Ag3 (agama); lane 8 – GA7 (gecko); lane 9 – GA2 (gecko); lane 10 – G10 (gecko); lane 11 – G9 (gecko); lane 12 – E4 (gecko); lane 13 – C3-1 (gecko). Molecular weight markers are placed in lane 1 (NEB 100bp ladder, sizes of selected bands are indicated on the left hand) and in line 7 (NEB 50bp ladder, sizes of selected bands are indicated on the right hand).

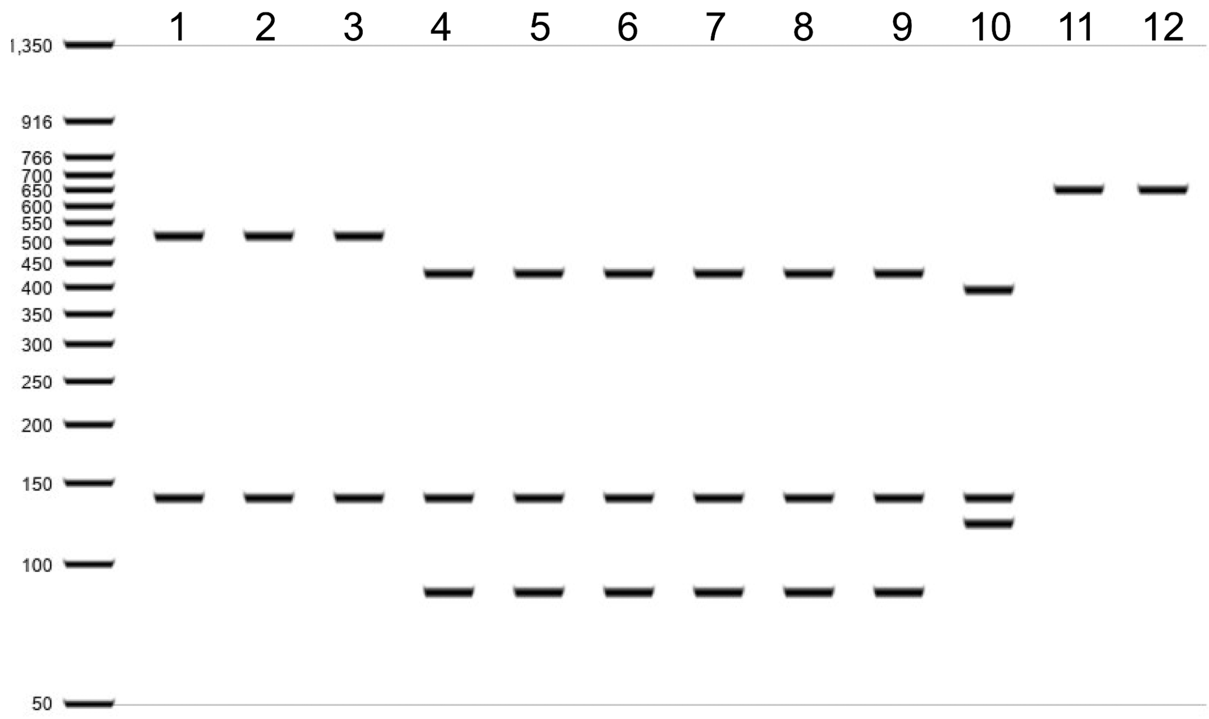

Figure S6

AccI in silico restriction pattern of eight sequences from human isolates and four representative *Basidiobolus* spp. isolates (GP8, Ag3, G10, E4) covering all four restriction patterns established in this study (all trimmed to the Ba1/Ba2 amplicon size; created with Geneious Prime Version 2020.2).

Lane 1 – *B.* sp. isolate GP8 (this study); reptile feces, South Africa
Lane 2 – *B. ranarum* strain IFM 41413 [AB363770.1]; subcutaneous granuloma, Thailand
Lane 3 – *B. haptosporus* strain NRRL 28635 [AF113451.1]; clinical isolate, Uganda
Lane 4 – *B.* sp. isolate Ag3 (this study); reptile feces, South Africa
Lane 5 – *B.* sp. isolate Doza [MH254938.1]; human gastrointestinal biopsy, Saudi Arabia
Lane 6 – *B*. sp. isolate 9-4 [MH256645.1]; human gastrointestinal biopsy, Saudi Arabia
Lane 7 – *B*. sp. isolate V81 [MH256648.1]; human gastrointestinal biopsy, Saudi Arabia
Lane 8 – *B*. sp. isolate F15-1 [MH256650.1]; human gastrointestinal biopsy, Saudi Arabia
Lane 9 – *B*. sp. isolate F43-5 [MH256651.1]; human gastrointestinal biopsy, Saudi Arabia
Lane 10 – *B.* sp. isolate G10 (this study); reptile feces, South Africa
Lane 11 – *B.* sp. isolate E4 (this study); reptile feces, South Africa
Lane 12 – *B. haptosporus* strain ARSEF 261 [EF392410.1]; 4-year old male, Indonesia
Numbers on the left hand indicate band sizes of a 50bp ladder.
